# Design, Fabrication, and Properties of High Damping Metal Matrix Composites—A Review

**DOI:** 10.3390/ma2030958

**Published:** 2009-08-18

**Authors:** Hui Lu, Xianping Wang, Tao Zhang, Zhijun Cheng, Qianfeng Fang

**Affiliations:** Key Laboratory of Materials Physics, Institute of Solid State Physics, Chinese Academy of Sciences, Hefei 230031, China; E-Mails: luhui@mail.ustc.edu.cn (H.L.); xpwang@issp.ac.cn (X.W.); zhangtao@issp.ac.cn (T.Z.); zjcheng@issp.ac.cn (Z.C.)

**Keywords:** metal matrix composite, high damping, internal friction

## Abstract

Nowadays it is commonly considered that high damping materials which have both the good mechanical properties as structural materials and the high damping capacity for vibration damping are the most direct vibration damping solution. In metals and alloys however, exhibiting simultaneously high damping capacity and good mechanical properties has been noted to be normally incompatible because the microscopic mechanisms responsible for internal friction (namely damping capacity) are dependent upon the parameters that control mechanical strength. To achieve a compromise, one of the most important methods is to develop two-phase composites, in which each phase plays a specific role: damping or mechanical strength. In this review, we have summarized the development of the design concept of high damping composite materials and the investigation of their fabrication and properties, including mechanical and damping properties, and suggested a new design concept of high damping composite materials where the hard ceramic additives exhibit high damping capacity at room temperature owing to the stress-induced reorientation of high density point defects in the ceramic phases and the high damping capacity of the composite comes mainly from the ceramic phases.

## 1. Introduction

The damping capacity of a material is an evaluation of the energy dissipated in the material during mechanical vibration. High damping materials, which possess the ability to dissipate mechanical vibration energy, are valuable in the application fields of noise control and for stabilizing structures to suppress mechanical vibrations and attenuate wave propagation [1,2,3,4]. Practical applications need low density materials that simultaneously exhibit a high damping capacity and good mechanical properties. However, in metals these properties are often incompatible, due to the dependence of the microscopic mechanisms involved in strengthening and damping. Therefore, it would be of interest to develop new materials that simultaneously exhibit good mechanical properties and high damping [3,5]. This is possible only when the microscopic mechanisms responsible for dissipation of the vibration energy are independent of that of the hardening and strengthening. Such a compromise can be achieved by the development of two-phase composites, in which each phase plays a specific role: damping or providing mechanical strength. Metal matrix composites (MMCs) are good candidates because firstly, MMC processing allows the possibility of tailoring the resultant damping properties by selecting high damping reinforcements, secondly, MMC processing modifies the microstructure of metals and alloys, thus introducing energy dissipation sources [1,6], and thirdly, typically hard and high strength reinforcements will improve the mechanical properties of the composites.

The damping behaviors of MMCs can be attributed to thermal mismatch-induced dislocation damping, interface damping, interaction damping and the rule of mixtures damping. Briefly, an enhanced dislocation density due to the thermal mismatch between reinforcements and matrix increases energy dissipation sources; the sliding of the interfaces between the reinforcements and matrix dissipates energy under cyclic loading; interactions between reinforcements and dislocations or grain boundaries may lead to changes in damping response, such as dislocation pinning or viscous sliding of grain boundaries; the intrinsic damping of the reinforcements may be independent of that of the matrix material, which leading to a rule of mixtures effect on the overall damping behavior [1,6,7].

In this review, the main achievements during the past decades were summarized, with an emphasis on the design, fabrication, characterization and performance of high damping MMCs. Finally, a new design concept of high damping composite materials is suggested.

## 2. Design of High Damping Metal Matrix Composites

Metal matrix composites (MMCs), like most composite materials, provide significantly enhanced properties compared to conventional monolithic materials, such as higher strength, stiffness, hardness, creep resistance, weight savings, etc. The strengthening mechanisms of MMCs may be divided into two categories: classical composite strengthening mechanisms and dislocation punching strengthening mechanisms. The former refers to the fact that the applied load is transferred from the softer matrix, across the matrix/reinforcement interface, to the typically higher stiffness reinforcements, which carry much of the applied load. The later refers to that the high thermal mismatch between the metallic matrix and the ceramic reinforcement induces dislocations in the matrix near the reinforcement/matrix interface upon cooling. The dislocation punching results in the strengthening of the matrix [8,9,10,11]. The design philosophy of high damping MMCs should give consideration to both the damping mechanism and the strengthening mechanisms.

The reinforcements in a composite can take either discontinuous (particulates, platelets, whiskers or chopped fibers) or continuous (typically long fibers) form. Continuous fiber reinforcements may provide the most effective strengthening (in a given direction), while particulate reinforced materials are more attractive owing to their cost-effectiveness, and isotropic properties. In this section, emphasis will be placed on the later. In view of the special properties of nano-scaled materials, the nano-scaled dispersoids reinforced MMCs will be discussed separately.

### 2.1. Discontinuously reinforced high damping MMCs

Discontinuously reinforced MMCs have attracted considerable attention because of their feasibility for mass production, promising mechanical properties and potential high damping capacity. In particular, discontinuously reinforced aluminum alloy MMCs provide high damping and low density and allow undesirable mechanical vibration and wave propagation to be suppressed [6].

Discontinuous reinforcements include carbides, nitrides, borides, oxides, and simple substance of carbon, among which SiC [12,13,14,15,16,17,18,19,20], Al_2_O_3_ [21,22,23] and graphite [12,24,25,26,27] particulates are the most frequently used. As an example, SiC, as derived from rice hulls, is relatively inexpensive and can be produced in large quantities [28]. Lavernia *et al.* [6] demonstrated that adding SiC or Al_2_O_3_ particulates into the aluminum matrix could provide substantial gains in specific stiffness and strength, while the resulting changes in damping capacity were either positive or negative. Graphite particulates could produce a remarkable increase in damping capacity, but at the expense of stiffness. Wei *et al.* [24,27] also pointed out that the damping capacity of the macroscopic graphite particulates reinforced pure aluminum composite is increased with a larger volume fraction of the reinforcements, however, this is accompanied with a decrease in dynamic modulus. Rohatgi *et al.* [29] investigated the damping capacity of graphite and silicon carbide particulate reinforced Al alloy composites. As expected, the damping capacity of graphite/Al alloy composites increased with the volume percentage of graphite within the range studied. However, no obvious improvements in damping capacity were observed by dispersion of silicon carbide in aluminum alloy. In contrast, Srikanth and Gupta [14] reported that the damping capacity of the pure magnesium matrix was improved in the presence of SiC particulates, and increased with the increase of the proportion of SiC particulates. SiC whisker reinforced MMCs have shown more attractive combinations of strength, fracture toughness, and thermal stability than the particulate-reinforced MMCs. However, extensive application of these MMCs has been limited as a result of production costs and health hazards. Moreover, from the standpoint of developing high damping materials, SiC whisker reinforced Al alloy MMCs do not exhibit improved damping capacity than that of unreinforced aluminum alloys [6].

Ceramic hollow sphere fly ash (FA) particulates (with enclosed porosities) is another kind of interesting reinforcement, which is inexpensive, low density and available in large quantities as solid waste by-product during combustion of coal in thermal power plants. Wu *et al.* [30] investigated the damping properties of the hollow sphere fly ash/6061Al composites. The damping capacity of the FA/6061Al composite can reach 3.2 × 10^−2^, which is in the high damping capacity level. Sudarshan and Surappa [31] also showed that fly ash/A356 MMCs, with proper volume fraction of fly ash particulates, exhibited improved ambient temperature damping capacity, as well as the hardness, elastic modulus, and 0.2% proof stress, compared to unreinforced alloy. However, the tensibility decreases, owing to the presence of pores and decrease in the volume fraction of ductile matrix.

**Figure 1:**
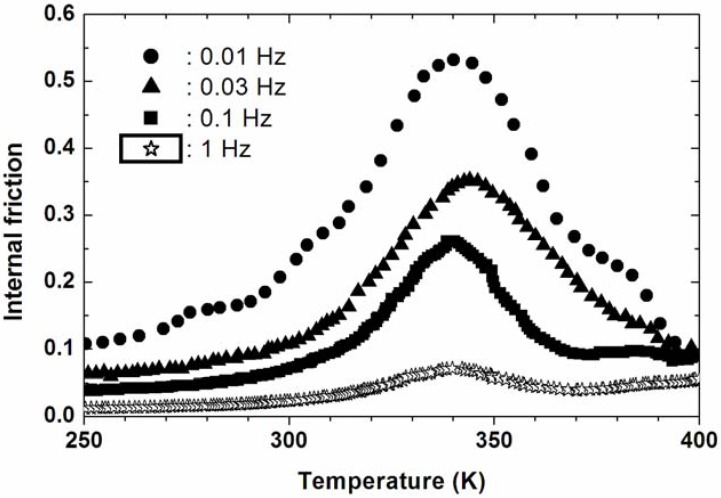
Internal friction spectra of Cu-Al-Ni particulates reinforced indium matrix composite at 0.01 Hz (circles), 0.03 Hz (triangles), 0.1 Hz (squares) and 1 Hz (stars) [40].

Shape memory alloys (SMAs) have attracted considerable attention due to their excellent damping capacity and high mechanical properties. The twin boundaries in martensite can be easily moved by an external stress to accommodate the strain, which is believed to be responsible for the high damping of TiNi based alloys [32]. It has been experimentally and analytically demonstrated that SMA particulates or fibers in a non-SMA metal matrix can enhance the damping capacity of the composite, as well as some other unique properties or functions such as self-strengthening, active modal modification, damage resistance and control [33,34,35,36,37,38]. For instance, Yamada *et al.* [33] proposed the concept to strengthen the aluminum MMCs by the shape-memory effect of dispersed TiNi SMA particulates. Because the martensite phase of SMAs has a comparatively high loss factor, an improvement in the damping capacity of the SMA particulate-reinforced composites is expected at the martensite stage. Consistent with the aforementioned work, Thorat *et al.* [39] showed that addition of NiTi particulates increased the damping capacity as well as the precipitation kinetics of AA2124 composites compared with the base AA2124. San Juan and No [40] developed a new kind of high damping metal matrix composites, based on powders of Cu–Al–Ni shape memory alloys (SMAs) embedded in an indium matrix. The damping is enhanced in all the temperature range, and especially at the peak (70 °C) reaches tan φ = 0.54 at 0.01 Hz. However, the damping decreases dramatically with increasing frequency, and reaches 0.07 at 1 Hz, as shown in Figure 1. Similar work has been done by them and their coworkers [41], with the alternative In-Sn alloy matrix. The value of damping (tan φ) is also more than 0.5 at low frequencies. The authors claimed that the high damping capacity was caused by the synergetic effect between the damping of the shape memory alloy and the damping of the matrix alloy. However, no mechanical properties were reported in their work and should be quite low owing to the soft In-Sn alloy matrix. On the other hand, the high damping capacity mainly coming from the shape memory alloys only occurs at the phase transformation temperature and decreases dramatically with increasing frequency, a stable damping capacity could not be expected in such composite materials.

Piezoelectric materials polarize along specific crystal directions and induce electric charges on the surfaces when stress is applied. It is expected that energy dissipation would occur by shunting the induced electric charges. Piezoelectric ceramics have been used in high damping MMCs, with the damping mechanism related to piezoelectric effects. Yoshida *et al.* [42] investigated the damping mechanism of metal-piezoelectric ceramic composites. The research indicated that piezoelectric ceramic PbTiO reinforced Cu composite exhibited three to five times higher damping capacity than the pure Cu.

The matrix materials of MMCs can be various, such as alloys based on aluminum, magnesium, iron, titanium, zinc, copper, zirconium, nickel, tin, lead, and other pure metals. Ferromagnetic alloy is another kind of damping material, which provides damping through the magnetomechanical mechanism (*i.e.,* movement of the magnetic domain boundaries during vibration). Specially, SMA and ferromagnetic alloy are used as the matrix of high damping MMCs, beyond the most frequently used aluminum and magnesium alloy. Graphite particulates/CuAlMn [43], magnetic powders/SMA matrix composites, such as Fe-Cr, Fe-Cr-Al and Fe-Al flake reinforced Cu-Zn-Al matrix, show a high passive-damping capacity [44].

As is well known, some mechanical properties of materials are dependent of the reinforcement geometry (particulates vs. whiskers) and orientation (perpendicular vs. parallel), which researchers have studied systematically [45]. However, an understanding of the correlation between material damping and the reinforcements has eluded investigators, partly as a result of the fact that various mechanisms are involved [1].

### 2.2. Nano-scaled dispersoids reinforced high damping MMCs

Nano-scaled reinforcements (particulates or whiskers) can increase the specific modulus and other mechanical properties of MMCs [46]. As an example, Peng and Zhu [47] showed that MMCs reinforced by combining nano-scaled dispersoids with micro-sized particulate or whisker reinforcements present improved mechanical properties, for nano-scaled dispersoids can remarkably increase the high temperature strength of MMCs. However, there is not much analytical and experimental work reported on the effect of nano-scaled reinforcements on the damping capacity of MMCs.

Kireitseu *et al*. [48] demonstrated that carbon nanotubes (CNT) employed to reinforce MMC were able to enhance structural damping as well as composite stiffness. Advanced mobility of nanotubes results in advanced strength and vibration damping properties in a wide range of frequency and temperature. Interfacial fracture energy between nanoparticle and matrix plays a great role for a total energy dissipated by the damping material. Yang and Schaller [49], however, pointed out that in the CNT reinforced magnesium matrix composites, the thermal mismatch at the interface in the CNT composites was greatly reduced owing to the size reduction of reinforcements and thus the transient damping was of lower intensity. Therefore, whether CNT could act as an appropriate reinforcement in high damping MMCs is still an open question.

Deng [50] showed that multi-walled carbon nanotubes (MWNTs) were a promising reinforcement for metal matrix composites to obtain high damping capabilities at an elevated temperature without sacrificing the mechanical strength and stiffness of a metal matrix. The damping capacity of MWNTs reinforced 2024Al composite reaches 0.0975 (which was wrongly written as 0.975 in the literature) and the storage modulus is 82.3 GPa at 0.5 Hz and 400 °C.

Nano-scaled Al_2_O_3_ particulate is another kind of researched reinforcement. Srikanth *et al.* [4] synthesized the nano-scaled Al_2_O_3_ particulate reinforced Mg matrix composites, and studied the damping capacity, elastic modulus and hardness, as listed in Table 1. They also agreed to the increase in damping with the presence of nano-scaled particulates, which can be attributed to simultaneous influence of various intrinsic and extrinsic damping mechanisms.

Nanotechnology could potentially be used to fabricate advanced materials with superior dynamic properties such as damping behavior at wider frequency and temperature ranges, as well as improved mechanical properties such as stiffness. However, the damping property of nano-scaled material is mostly unknown and requires extensive investigations.

**Table 1:** Damping capacity, elastic modulus and hardness of Mg and nano-Al_2_O_3_/Mg [4].

Wt.% of Al_2_O_3_	Loss factor η	Dynamic modulus (GPa)	Hardness (HV)
0	0.0085	39.52	39.6 ± 2.1
1.0	0.0115	41.29	52.3 ± 1.1

### 2.3. Fiber reinforced high damping MMCs

As mentioned in the previous section, SMA fibers, as well as particulates, can be used as reinforcements of MMCs, resulting in enhanced damping capacity, as well as some other properties. For instance, the damping capacity of the TiNi fiber Al matrix composite was measured by using a uniaxial vibration method, and the results indicated that the damping capacity (tan δ) of the composite in the temperature range of 270-450 K was substantially improved over the unreinforced aluminum [51]. It was found that the factors such as SMA fiber volume fraction, the fiber orientation and the stacking sequence of SMA composites have strong influences on the specific damping capacity (SDC) and on the natural frequencies of the SMA hybrid composites and structures [37].

Mayencourt and Schaller [5] have found that Mg-2 wt% Si alloys reinforced with long carbon fibers exhibited a specific Young’s modulus of about 200 GPa with a damping capacity of 0.01 at a strain amplitude of 10^-5^. They also studied the high damping magnesium matrix reinforced with Mg_2_Si fibers and draw a conclusion that the mechanical strength can be optimized without reducing the damping capacity. In their research, the Mg_2_Si/Mg composites were processed by unidirectional solidification of Mg-2 wt% Si alloy. The c-axis orientation of pure magnesium matrix provided a high damping capacity over a wide range of temperatures and the unidirectional fibers enhanced the mechanical strength up to that of some industrial magnesium cast alloys such as AZ63. Since these mechanisms are independent, the high damping capacity and the high mechanical strength can be reached in these composites, without regard of the complex interaction between dislocations and point defects [52].

Recently, however, Chowdhury *et al.* [53] found that in the carbon fiber reinforced magnesium based MMCs, with the metallic matrix unidirectionally oriented with respect to the matrix-reinforcement interface by unidirectional solidification technology and metallic matrix without orientation by infiltration method only, the damping was lower in the orientated specimen than in the as infiltrated one in all the temperature range. In the former case, the basal slip plane is nearly perpendicular to the interface normal, strongly reducing the coupling between the thermal stress along the interface and the mechanical stress from the torsion test. Meanwhile, some researches indicate that the damping behavior of continuous graphite fiber reinforced magnesium alloy is inferior to the unreinforced MMC [54]. As a summary, the design concepts, targets, and deficiencies for some MMCs are listed in Table 2, while the details of Li_5_La_3_Ta_2_O_12_ additives and the related MMCs will be described in Section 5.

**Table 2:** Design concepts, target, and deficiencies for some MMCs.

Reinforcements in MMC	Design concepts (aim at reinforcements)	Targets	Deficiencies
SiC, Al_2_O_3_	Make use of the high stiffness and strength	Improved specific stiffness and strength	Whether the damping capacity can be improved is ambiguous
Graphite	Make use of the high damping capacity	Improved damping capacity	Deteriorated stiffness
Fly ash	Make use of the hollow ceramic structure	Improved ambient temperature damping, hardness, stiffness	Deteriorated tensibility
SMAs	Make use of the high damping capacity and mechanical properties	Improved damping capacity and some functions as SMA has	High damping capacity only occurs at the phase transformation temperature and decreases dramatically with increasing frequency
Piezoelectric ceramics	Make use of piezoelectric effects	Improved damping capacity	Related research is deficient
Nano-scaled dispersoids	Make use of the effect of particulate size	Improved mechanical properties	Whether the damping capacity can be improved is ambiguous
Li_5_La_3_Ta_2_O_12_	Make use of the damping mechanism of point defects, and the hardness of the ceramics	Improved damping capacity and mechanical performance at room temperature	Further study is underway

## 3. Fabrication of High Damping MMCs

Traditional processes for fabricating MMCs include powder metallurgy, spray deposition, mechanical alloying and various casting techniques [8,55,56,57,58,59,60], which are also used in the case of high damping MMCs, with a wide range of matrix materials (aluminum, magnesium, titanium, copper, nickel and iron) and reinforcements (carbides, nitrides, borides, oxides and carbon). In the following subsection, we only give an overview and outline the chief features of the main methods.

Ibrahim *et al.* [8] suggested that the processing methods utilized to manufacture particulate reinforced MMCs can be grouped according to the temperature of the metallic matrix during processing, such as liquid phase processes, solid state processes, and two phase processes. Here, we follow this categorization to classify methods for the fabrication of various high damping MMCs.

### 3.1. Solid state processes

The powder metallurgy (PM) process involves steps including: powders sieving, blending, pressing, degassing and consolidation. The PM methods have been successfully applied to a large number of metal/ceramic combinations. In terms of microstructural requirement, the PM approach is superior in view of the rapid powder solidification. This allows the development of novel matrix materials outside the compositional limits dictated by equilibrium thermodynamics in conventional solidification processes. The main deficiencies of this process are complex processing, relatively high cost and hard controlling. As a variant of PM, powder injection molding (PIM) is a near net-shape manufacturing technology that combines the shaping effect of plastic injection molding with the potential of powder metallurgy for working over metal and ceramic powders [61,62]. The process involves four steps: mixing, injection molding, debinding (dewaxing) and sintering. One of the evolutional variations of the PIM technology is metal injection molding. It is applicable for the fabrication of composites with discontinuous reinforcements, typically particulates, whiskers or short fibers. The process offers advantages for the mass production of small and complex parts such as low production cost, near net-shape, geometric complexity, good tolerance and reproducibility. Additionally, it can control the orientation of the fibers when fabricating short fibers reinforced MMCs. However, this process suffers from obstacles including the need for fine spherical powders and proper selection of binder [63]. High damping MMCs have been fabricated by PM, for instance, Fe-Cr flakes/Cu-Zn-Al composites [44] and nano-Al_2_O_3_/Mg [4].

### 3.2. Liquid phase processes

Liquid phase processes refers to that the reinforcements are incorporated into a molten metallic matrix using various fabrication techniques, including liquid metal-discontinuous reinforcements mixing, melt infiltration and melt oxidation processes. For the basic aspects of the solidification and casting techniques, the reader can refer to [64].

In liquid metal-discontinuous reinforcements mixing process, it is necessary either adding wetting agents to the melt or coating the ceramic particulates prior to mixing, for most ceramic materials cannot be wetted by the molten alloys. In this process, a strong bond between the matrix and the reinforcement is formed owing to the high processing temperatures. This process has reached an advanced stage of development, while some difficulties exist, including: agglomeration of the ceramic particulates during agitation, settling of particulates, segregation of secondary phases in the metallic matrix, extensive interfacial reactions and particulate fracture during mechanical agitation. High damping MMCs have been made by using this method or variants of it (disintegration melt deposition), such as Al_2_O_3_/Al alloy [21,22,65], graphite/Al alloy [29], SiC/Al alloy [29], SiC_p_/Al-Li [17], SiC/Mg [18], fly ash/Al alloy [31].

In melt infiltration processes, a molten alloy is introduced into a fiber preform or a porous ceramic, utilizing either inert gas or a mechanical device as a pressurizing medium. This approach has been commercialized. Some of the drawbacks of this process include reinforcement damage, coarse grain size and undesirable interfacial reactions. Various high damping MMCs have been made by using this method, such as TiNi/Al [51], SiC/Mg-2-wt%-Si [5], C/Mg-2-wt%-Si [5,66], graphite/Al [24], (SiC_p_+Al_2_O_3_·SiO_2f_)/Mg [15], SiC/Al [19,67], SiC/Mg [67], Al_2_O_3_/Mg alloy [23], graphite/Zn-Al alloy [27], and carbon fiber/Mg [53].

### 3.3. Two-phase processes

In the spray deposition process, the reinforcement particulates are introduced into the stream of molten alloy which is subsequently atomized by jets of inert gas and collected on a substrate in the form of a reinforced metal matrix billet. Products with excellent properties can be synthesized using this method. However, the main drawback of this method is high cost. High damping MMCs have been made by using this method, such as SiC, graphite or both of them reinforced Al or Al alloy, Mg or Mg alloy [6,12,16,25,68].

Variable co-deposition of multi-phase materials (VCM) processing is another two-phase process. The matrix metal is disintegrated into a fine dispersion of droplets using high velocity gas jets. Simultaneously, one or more jets of strengthening phases are injected into the atomized spray at a prescribed spatial location where the droplets contain a limited amount of volume fraction of liquid. Hence, contact time and thermal exposure of the particulates with the partially solidified matrix are minimized, and can be controlled. In addition, tight control of the environment during processing minimizes the oxidation of the matrix.

## 4. Damping Behavior of Al, Mg Based MMCs

Light weight metallic materials such as Al and Mg, are getting popular in the design of dynamic mechanical systems in semiconductor equipments, aerospace and defense related industries. Mg is more preferred for its low density of 1.74 g/cc than Al (2.7 g/cc), while the static stiffness of magnesium, measured in terms of elastic modulus, remains at about 40 GPa, which is well below that of Al (70 GPa) [69]. Hence Mg and Al have similar specific stiffness [4]. In addition, magnesium possesses better damping characteristics than any other light weight metals, which helps in dissipating the stored strain energy in the components [14]. This section will focus on Mg and Al matrix composite with further improved inherent damping capacity and stiffness that will promote their usage in various engineering applications.

The damping properties are deduced from mechanical spectroscopy measurements. Quantitative comparison of the damping capacity of materials is difficult, because of the differences in testing method (torsion pendulum methods [5,21,24,27,30,39,42,52,53,67], suspended beam method [4,14,16,18,29], dynamic mechanical thermal analyzer technique [12,15,17,19,22,25,31], Piezoelectric ultrasonic composite oscillator technique), exciting mode (impulse excitation [4,16,18], forced vibration [24,27,30,67], free decay [5,23,39], resonant vibration [30]), testing parameters (temperature, the loading frequency, strain amplitude) [70,71], and specimen configuration (wire, rod, sheet). On the other hand, measures of damping capacity include loss angle (φ), loss tangent (tan φ), inverse quality factor (Q^-1^), loss factor (η), logarithmic decrement (δ) and specific damping capacity (SDC, ψ). They are interchangeable with a proper conversion in cases of relatively small damping capacity (tan φ < 0.1) by the following equation:

φ ≈ tan φ = Q^-1^ = η = ψ/2π ≈ δ/π (1)

### 4.1. Damping behavior and mechanisms in Al, Al alloy and Al based MMCs

In general, Al alloys exhibit a relatively low damping capacity. MMC techniques provide modifications to the matrix microstructure including reinforcement/matrix interfaces and dislocations in the matrix induced by thermal mismatch strain. Damping capacities of some frequently used particulate reinforcements, such as graphite (Gr), Al_2_O_3_, SiC, and Si_3_N_4_, are listed in Table 3.

Zhang *et al.* [1,3,6,12,68] fabricated damping MMCs with Al alloy matrix and SiC, Al_2_O_3_ and graphite (Gr) particulates reinforcements. In a direct view, Table 4 lists typical values of damping capacity of the MMCs at 50 °C and 250 °C, respectively. It can be seen that both SiC/2519Al and Al_2_O_3_/2519Al MMCs show a similar damping capacity as that of the unreinforced 2519Al around 50 °C, but a higher damping capacity than that of the unreinforced 2519Al around 250 °C. The high damping phenomena at high temperature may be ascribed to thermally activated grain boundary damping and interface damping. The largest gains in damping capacity were observed in the Gr/2519Al MMCs, which was 1.5 times as high as that of the unreinforced 2519Al in the testing temperature range. The damping mechanisms in the Gr/2519Al MMCs were thought to be the intrinsic damping of graphite particles, the dislocation damping, and the interface damping. The addition of graphite to aluminum alloys is not favorable for mechanical properties. In proper ratio of SiC and graphite, the SiC_p_/Gr/Al composite may possess high damping capacity, as well as fine mechanical properties.

**Table 3:** Damping capacity of graphite, Al_2_O_3_, SiC, Si_3_N_4_, and Li_5_La_3_Ta_2_O_12_.

Material	Test	T (°C)	f (Hz)	Maximum loss factor	Ref.
Graphite	Bending	30-250	0.1-10	0.013	[68]
A1_2_O_3_	Axial	0-1200	-	0.0005	[72]
SiC	Torsion	20-1400	10-15	0.0016-0.003	[73]
Si_3_N_4_	Torsion	20-1250	10-15	0.003	[73]
Li_5_La_3_Ta_2_O_12_	Torsion	20-250	0.1-10	0.11	[74]

**Table 4:** Typical values of damping and modulus of 2519Al and MMCs at 1 Hz [68].

Material	tanφ	
	50 °C	250 °C
As received 2519	0.005	0.008
Spray deposited 2519	0.005	0.014
SiC/2519Al	0.004	0.020
Al_2_O_3_/2519Al	0.003	0.018
2922Gr/2519Al	0.008	0.019

**Figure 2:**
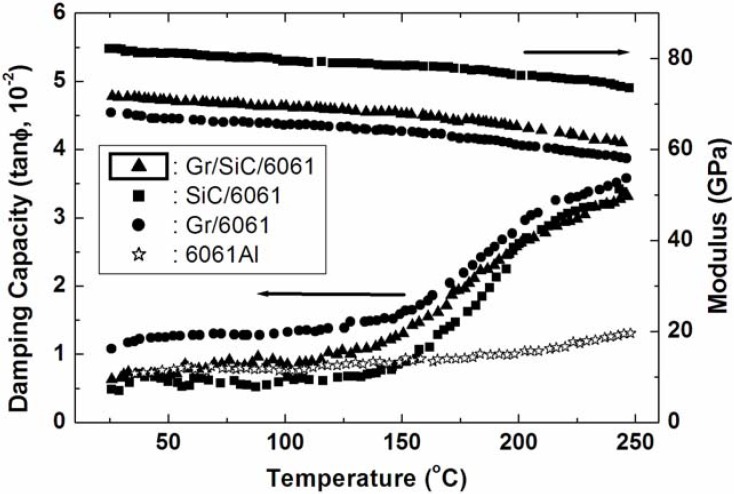
Damping capacity of SiC/Graphite/6061Al, SiC/6061Al, Graphite/6061Al MMCs and 6061Al [12,75].

In 1997 the research group in University of California proposed a high damping composite of SiC/Graphite/6061Al [75]. Figure 2 reveals that the damping behavior of SiC/Graphite/6061Al is dominated by the presence of graphite particles while the stiffness of SiC/Graphite/6061Al is primarily determined by the presence of SiC particles. Taking the SiC/Graphite/6061Al/21p (V_SiC_=10.5% and V_Gr_=10.5%) as an example, after extrusion and heat treatment, the yield strength increased from 92 MPa to 280 MPa while the room temperature damping capacity (Q^-1^) was still at the level of ~0.01. Since the matrix 6061Al alloys showed the strength of 249 MPa at the same treatment, the strengthening effect of the reinforcement particles were quite small. From their results the interface damping mechanism in such composite (with extrusion and heat treatment) cannot be expected, and therefore a reinforcement phase with both high damping capacity and strengthening effects are necessary for the purpose of high damping composite materials.

### 4.2. Damping behavior and mechanisms in Mg, Mg alloy and Mg based MMCs

Magnesium has a remarkable dislocation damping effect, which refers to a well-defined amplitude-dependence of the internal friction and Young’s modulus. The dislocation damping effect is caused by the movement of dislocations, which are weakly pinned on the basal plane. High damping magnesium alloys base on the dislocation damping effect [76,77]. The Mg-Al and Mg-Zn type of magnesium alloys offer good combination of room-temperature strength and ductility, and good salt-spray corrosion resistance, while magnesium alloys consisted of rare-earth, alkaline earth or silicon possess good performance at elevated temperature [78].

Studies showed that addition of hard reinforcing particulates in a ductile metallic matrix helps to improve the overall damping capacity and stiffness. As a typical instance, Srikanth *et al.* [18] investigated the effect of presence of SiC particulate reinforcement in Mg matrix. In these MMCs, the hard ceramic particulates cause high residual stresses around the particulates in the form of an annular plastic zone, owing to a large difference in the coefficient of thermal expansion of magnesium and silicon carbide. According to the model about plastic zone damping proposed by Carreno-Morelli *et al.* [21], the damping depends directly on the volume fraction of the plastic zone and the strain amplitude, hence the damping of the pure magnesium matrix increased as a result of the presence of SiC particulates. A comparison among representative materials of high damping Al or Mg based MMCs reported in the latest three years is shown in Table 5.

**Table 5:** Damping data of Al or Mg based MMCs at room temperature in the latest three years.

Material	Reinforcement volume fraction	Loss tangent tanφ	f (Hz)	Test	Mechanical property		Ref.
Fly ash/A356	0	0.018	10	DMA	Dynamic Young’s modulus (GPa)	69	[31]
	6 vol.%	0.03				70	
	12 vol.%	0.04				74	
TiAl_3_/Al	0	0.004	1-10	DMA	-		[79]
	5 wt.%	0.008					
	10 wt.%	0.012					
FeAl_3_/Al	0	0.005	0.5	DMA	-		[80]
	5 wt.%	0.01					
TiC/AZ91	0	0.012	0.1	DMA	Young’s modulus (GPa)	45	[81]
	8 wt.%	0.020				49.1	
C_fiber_/Mg-2 wt.%Si	-	0.01	1	torsion pendulum	Shear modulus (GPa)	18.8	[82]
Li_5_La_3_Ta_2_O_12_/Al	0	0.0027	2	torsion pendulum	Compressive strength (MPa)	95.4	[83]
	20 wt.%	0.0098				136.2	
	25 wt.%	0.018				-	

## 5. A New Design Concept of High Damping MMCs

In general, the design problem of high damping materials is how to utilize the damping mechanisms associated with the stress-induced movements of crystalline defects, including point defects, dislocation and planar defects. Especially, the dislocation and planar defects (such as grain boundaries and domain walls) are more widely utilized owing to their relatively high specific damping level. In this case however, exhibiting simultaneously high damping capacity and good mechanical properties has been noted to be normally incompatible because the dislocation and planar defects are also the parameters that control mechanical strength. In this sense the damping mechanism of point defects will not deteriorate the mechanical properties while enhances the damping capacity of the materials. More importantly, the usually applied reinforcement phase, such as Al_2_O_3_, SiC, and TiB_2_ etc. that exhibit low intrinsic damping capacity, could not bring to the composite a high damping behavior at room temperature since the dislocation damping and interface damping should become noticeable only at quite high temperatures. It becomes clear that a reinforcement phase with a quite high damping capacity and the matrix alloys that supply satisfactory configuration to the inclusion particles are necessary for a smart high strength high damping composite materials design.

Instead of dislocations and planar crystalline defects, the damping mechanism of point defects, which enhances also the mechanical properties of materials, has been emphasized recently. For example, Yin *et al.* [84] invented a new kind of high temperature damping alloy based on the body-centered cubic β-type Ti alloys. The high damping capacity (as high as Q^-1^=0.08 at 250 °C) was realized through the Snoek-type relaxation process of high concentration interstitial oxygen atoms and the yield strength of the alloy was also obviously increased with the solute strengthening effects of the interstitial oxygen atoms.

**Figure 3:**
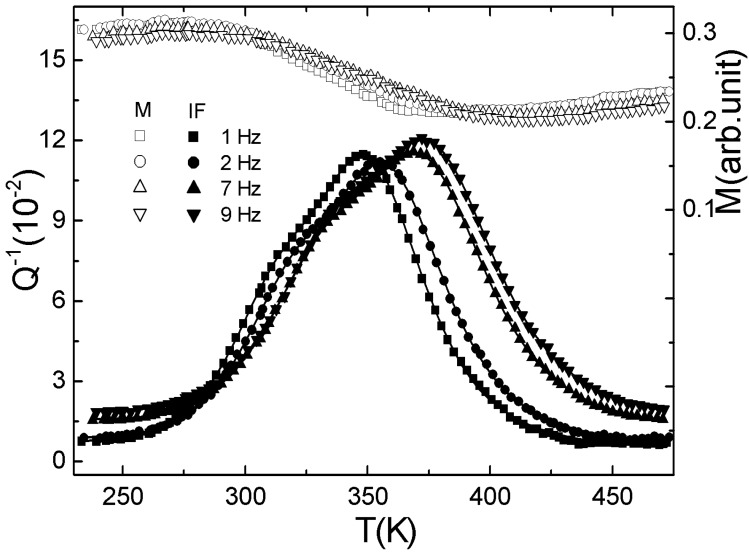
The temperature dependence of internal friction (*Q*^−1^) and relative shear modulus (M) for a Li_5_La_3_Ta_2_O_12_ compound [74].

Wang *et al.* [74] discovered the giant damping capacity in Li_5_La_3_Ta_2_O_12_ based oxides during the crystalline defects studies in the lithium ionic conductive ceramics. Li_5_La_3_Ta_2_O_12_ based oxides with the garnet like structure are a novel family of fast lithium ionic conductors and the lithium ionic conductivity is as high as ~4×10^-5^ S/cm at room temperature. Related to the high conductivity of lithium ions, the plenty of lithium vacancies [85] in Li_5_La_3_Ta_2_O_12_ crystals result in the high damping capacity for such oxide ceramics through the stress-induced reorientation of the high concentration lithium vacancies (Snoek-type relaxation). The latest experimental results showed that the maximum damping value (Q^-1^) of the Snoek-type relaxation in the Li_5_La_3_Ta_2_O_12_ ceramics can reach as high as 0.11 at 350 K and 1 Hz [74], as presented in Figure 3, which is about three orders higher than that in other ceramics (such as Al_2_O_3_, SiC, TiB_2_). It should be noted that the maximum damping shifts toward higher temperature with increasing frequency but hardly changes the damping level, which is quite different from the damping caused by the phase transition. It is more interesting that the activation energy for Li^+^ to move can be easily varied with composition adjustment of the oxides. The variable activation energy for Li^+^ movement indicates that we can control the damping peak temperature very easily to sustain the temperature stable damping behavior. According to the reported data activation energy for Li^+^ movement in such oxides can be varied from 0.5 eV to 1.2 eV, which indicates that a high damping peak can be obtained in a large temperature range from 250 K to 523 K when the composition of the oxide is adequately adjusted [86,87].

**Figure 4:**
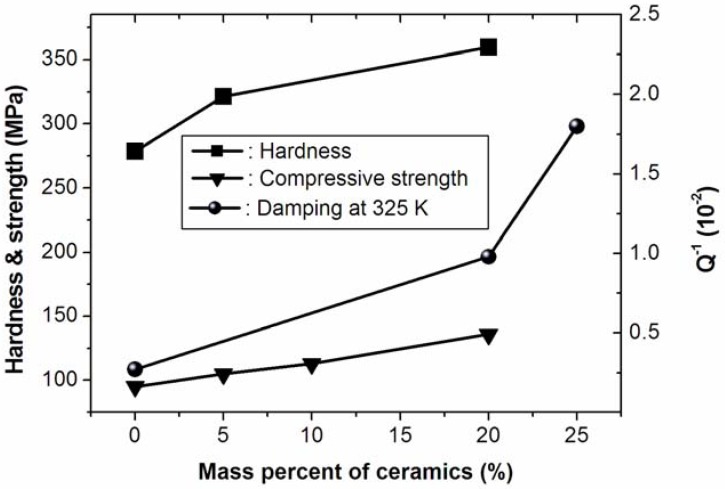
Ceramic content dependence of the internal friction (*Q*^−1^), hardness, and compressive strength for Li_5_La_3_Ta_2_O_12_/Al MMCs [83].

A novel concept to design metal matrix composites that exhibit high damping capacity as well as good mechanical performance at room temperature was proposed by Fang *et al.* [88]. The oxide particulates, which possess high damping capacity and high hardness, are dispersed into the high strength metallic alloys to form the MMCs. The oxide particulates are expected to contribute not only to the enhancement of damping capacity owing to their high intrinsic damping level, but also to the strength enhancements owing to the mechanism of particulate strengthening. Wang *et al.* [83] recently confirmed this design concept with Li_5_La_3_Ta_2_O_12_/Al composites prepared by powder metallurgy method. The damping capacity (Q^-1^) is observed to be 0.018 at 325 K and 2 Hz, as shown in Fig. 4 [83], and the yield strength and hardness of a Li_5_La_3_Ta_2_O_12_ (20%)/Al composite is about 40% and 30% higher than that of the pure Al, respectively. Therefore, through microstructure optimization and the fabrication route adjustment, high strength (>500 MPa) and high damping capacity (Q^-1^>0.03) composite materials could be realized with this novel design concept of MMCs.

## 6. Conclusions

The literature reviewed in this paper presents a cross-section of views and experimental results obtained by investigators in the field of high damping metal matrix composites (MMCs). The damping mechanisms of the MMCs are the intrinsic damping of the metal matrix, the intrinsic damping of the reinforcing particulates, and the particulate/matrix interface damping. Regarding the processing of high damping MMCs, a variety of classic techniques and variants can be utilized. Due to the incompatibility of high damping and good mechanical property, and the flexibility of damping mechanisms, most of the existing materials still cannot meet the demands. A new concept to design high damping MMC was proposed, taking advantage of the point defect damping in the ceramic additives, and further study is underway.
